# Washout Duration of Prostaglandin Analogues: A Systematic Review and Meta-analysis

**DOI:** 10.1155/2018/3190684

**Published:** 2018-09-27

**Authors:** Vlad Diaconita, Matthew Quinn, Dania Jamal, Brad Dishan, Monali S. Malvankar-Mehta, Cindy Hutnik

**Affiliations:** ^1^Ivey Eye Institute, London, ON, Canada; ^2^Schulich School of Medicine and Dentistry, Western University, London, ON, Canada; ^3^Queen's University, Ophthalmology Department, Kingston, ON, Canada; ^4^King Abdulaziz University, Saudi Arabia; ^5^St. Joseph's Health Care, London, ON, Canada; ^6^Department of Epidemiology and Biostatistics, Schulich School of Medicine and Dentistry, Western University, London, ON, Canada

## Abstract

**Topic:**

Prostaglandin analogues (PGAs) are first-line medical therapy for primary open angle glaucoma (POAG) and ocular hypertension (OHT). Intraocular pressure (IOP) lowering effects in full responders are known to be 25–33% for this class; however, partial responders and nonresponders do exist. In clinical trials or prospective series, discontinuation and washout of PGAs is necessary to evaluate true change in IOP from novel surgeries and medical therapies.

**Clinical Relevance:**

To identify all relevant papers with pertinent data on washout of PGAs and quantify the duration and long-term effect of reported PGA washout periods in glaucoma and OHT patients.

**Methods:**

A systematic review and meta-analysis was conducted to investigate the long-term effects on IOP after discontinuation of topical PGAs POAG and OHT patients. The main search was conducted in MEDLINE/PubMed, EMBASE, Cochrane Library, CINAHL, Web of Science, and BIOSIS Previews and conference proceedings.

**Results:**

1055 papers were identified, 548 were independently screened by two physicians., and 56 papers were analyzed for washout durations. The mean washout was found to be 4.56 weeks (±1.25), with the mode and median being 5 weeks. Five studies were analyzed as randomized control trials in which latanoprost was discontinued for 4 weeks prior to restarting another intraocular pressure-lowering drug. Meta-analysis revealed a 4-week discontinuation of latanoprost, on average, subjects returned to their baseline IOP.

**Conclusion:**

A significant IOP-lowering effect of latanoprost was not observed beyond 4 weeks, suggesting this may be an appropriate washout period for latanoprost. We could not identify appropriate washout periods for either travoprost or bimatoprost, although a majority of articles had 4-week washout durations for the two drugs. Despite the widespread use of this class of medication, there is a paucity of literature on the effects of PGA washout in patients that are treatment naïve to other topical medications.

## 1. Background

Intraocular pressure (IOP) is the only known modifiable risk factor for glaucoma. In the mid 1990s, prostaglandin analogues (PGAs) were introduced and are now recognized as first-line topical medical therapy for primary open angle glaucoma and ocular hypertension [1–3]. In Canada, latanoprost (generic and Xalatan, Pfizer, New York, NY, USA), travoprost (generic and Travatan, Alcon, Fort Worth, TX, USA), and bimatoprost (generic and Lumigan, Allergan, Irvine, CA, USA) are the widely available PGAs. IOP-lowering effects are known to be in the 25–33% range for this class, mostly by increasing uveoscleral outflow and minimally by increasing trabecular meshwork outflow [4–13] 

Jampel and colleagues performed a retrospective analysis of discontinuation of IOP-lowering medication. They showed that the largest change in IOP was observed after discontinuation of the first medication (33%) as compared to the second medication (9%) or third (13%). This difference was not class dependent. They concluded that the effectiveness of IOP-lowering medications may be significantly lower than that in ideal conditions [14]. The PGA class is dosed once daily, and during clinical trials, the typical washout period for prostaglandin analogues varies between 2 weeks and 8 weeks, with most trials using a 4-week washout. Lingering effects of medications beyond a presumed washout period could lead to erroneous conclusions about the efficacy and responder rates to subsequent medications, laser trabeculoplasty, minimally invasive, and/or filtering surgeries either alone or combined with cataract extraction. This would be true for both clinical practice as well as in clinical trials designed to evaluate new IOP-lowering drugs and devices.

It is for these reasons that the purpose of this study was to investigate if sufficient evidence exists to determine the long-term effects on IOP after discontinuation of topical PGAs in glaucoma or ocular hypertension patients (OHT). A systematic review and meta-analysis was conducted of the available literature.

## 2. Methods

### 2.1. Search Strategy

The effects of prostaglandins on the eye were first reported in 1985 by Giuffre [15]. The review therefore considered studies published in English between 1985 and 2016. Since clinical prostaglandin analogue use began after 1996, most papers were published between 1996 and 2016.

A preliminary search was conducted in MEDLINE to identify relevant MeSH terms and keywords. Then, a comprehensive search was completed using identified index terms and keywords and was adapted accordingly to different databases. The main search was conducted in six (6) databases: MEDLINE/PubMed, EMBASE, Cochrane Library, CINAHL, Web of Science, and BIOSIS Previews. Conference proceedings were included as found in EMBASE and Web of Science. The main search was conducted in May 2014, with a follow-up search on the years 2014–2016 conducted in October 2016. A medical librarian, experienced in systematic review searching, designed the search strategy and conducted the searches. The reference lists of included studies were searched to identify additional studies that may meet inclusion criteria.

Index terms used in the search were (adjusted by database) open angle glaucoma, Primary glaucoma, Ocular hypertension, Intraocular hypertension, Intraocular pressure, and prostaglandins.

Keyword terms used in the search were open angle glaucoma, POAG, OAG, (intra)ocular hypertension, Intraocular pressure Discontinu^*∗*^, stop^*∗*^, withdraw^*∗*^, washout, prostaglandins, bimatoprost, travoprost, latanoprost, and tafluprost.

### 2.4. Systematic Review

**Figure 1:**
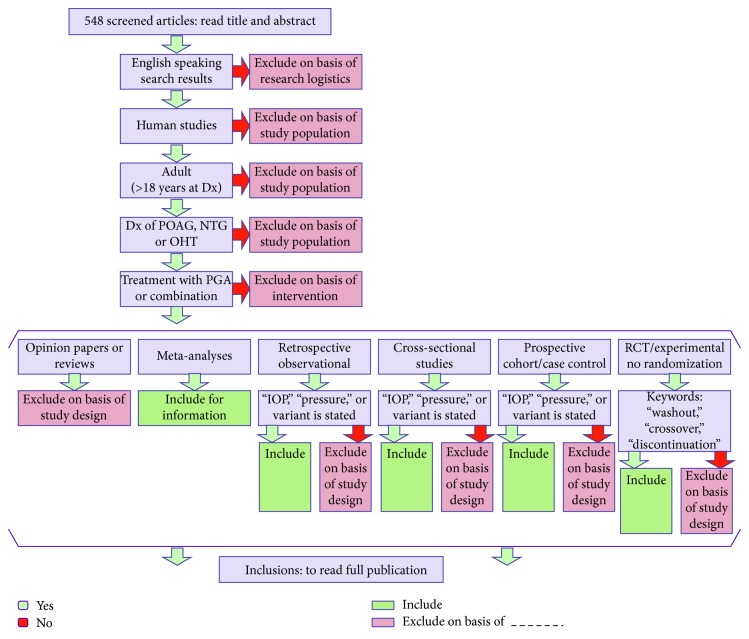
Inclusion and exclusion algorithm for screening articles.

Title screening was performed by two independent reviewers familiar with systematic reviews. Papers that were agreed upon by both reviewers were included in the next phase of analysis. Disagreements were reanalyzed by both reviewers, and agreement was reached by consensus. The inclusion/exclusion criteria are listed in Table 1 and the algorithm is shown in Figure 1.

### 2.5. Quality Assessment of Included Articles

All included articles were scored for quality using the Downs and Black checklist [16]. A quality check was performed to ensure completeness of our methodology.

### 2.6. Statistical Analysis

The primary outcome was the mean and standard deviation (SD) of pre- and postwashout IOP. Meta-analysis was completed on the primary outcome of interest using STATA v. 15.0 (STATA Corporation, College Station, TX). The extracted mean of the IOP at baseline and end point was used to compute the mean IOP reduction (IOPR) and percentage of IOP reduction (IOPR%) using the equations below [17]:

(1) $$\begin{array}{c} \text{IOPR}={\text{IOP}}_{\text{baseline}}-{\text{IOP}}_{\text{endpoint}}, \end{array}$$

(2) $$\begin{array}{c} \text{IOPR}\%=\frac{\text{IOPR}}{{\text{IOP}}_{\text{baseline}}}. \end{array}$$

For continuous scale outcomes such as mean values, standardized mean difference (SMD) was calculated as the treatment effect or effect size. SMD was chosen as the treatment effect since it is a mean difference standardized across all studies. To compute SMD for each study, the difference between the mean pre- and postoperative values for outcome measure (i.e., IOP) was divided by the SD for that same outcome measure. Weights were assigned to each SMD according to the inverse of its variance, and then average was computed. SMD for each study was then aggregated using the fixed- or random-effect model based on the presence of heterogeneity to estimate the summary effect.

To test heterogeneity, *I*^2^ statistics, *Z*-value, and *χ*^2^ statistics were computed. An *I*^2^ value of less than 50% implies low heterogeneity, and in these cases, a fixed-effect model was computed. An *I*^2^ statistics of 50% or more represents high heterogeneity, and in these cases, a random-effect model was calculated. Additionally, a high *Z*-value, a low *P* value (<0.01), and a large *χ*^2^ value imply significant heterogeneity, and therefore, a random-effect model using DerSimonian and Laird methods was computed. A forest plot was generated to display the statistics. A funnel plot was generated to check publication bias.

## 3. Results

### 3.1. Search Results

**Figure 2:**
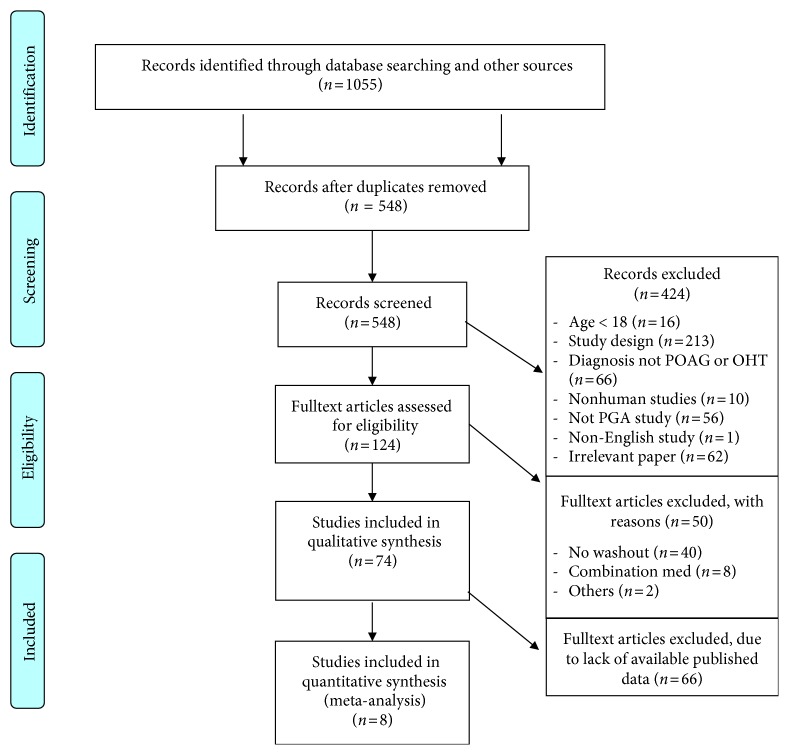
Prisma 2009 flow diagram.

1055 papers that met the search strategy were identified. 507 were removed as duplicates. The remaining 548 records were screened according to their title and abstract by two independent physicians. 424 were removed through the screening process. 213 were excluded due to study design: 66 were studies without a diagnosis of POAG or OHT, 62 were irrelevant papers to our search, 56 did not have prostaglandin analogue medications in the study protocol, 16 had an enrolment age of less than 18 years, 10 were nonhuman studies, and 1 was a non-English study without available translation (Figures 1 and 2).

**Figure 3:**
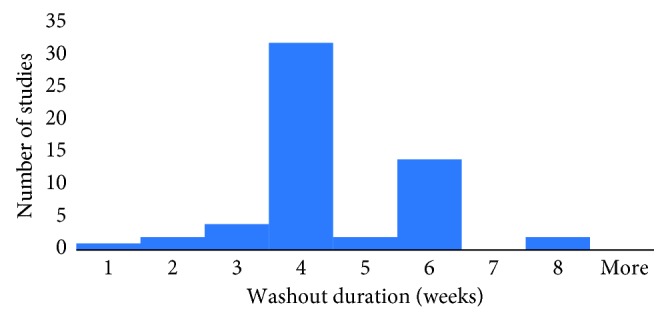
Washout duration of prostaglandin analogues and prostamides in research studies 1996–2016 (*n* = 56). Mean = 4.56 weeks; SD = 1.25; median = 4 weeks; mode = 4 weeks.

In total, 56 of the 74 papers which were analyzed listed washout durations for the prostaglandin analogues. Of the 56, 32 had a washout period listed of 4 weeks (57.1%). Seven had a washout of less than 4 weeks (12.5%), and 19 had a washout period of more than 4 weeks (33.3%) of which most were washouts of 6-week duration. The mean was 4.56 weeks with a standard deviation of 1.25 weeks. Both the median and mode washout period was 4 weeks (Figure 3).

### 3.2. Study Characteristics

Eight studies were identified that reported both means and standard errors/standard deviations for intraocular pressure. All eight studies were prospective, of which five were randomized control trials in which latanoprost was discontinued for 4 weeks prior to restarting another intraocular pressure-lowering drug. For each of those five papers, IOP was measured and documented prewashout (on latanoprost) and 4- week postwashout (off latanoprost). Each study had differing inclusion and exclusion criteria. Overall, studied participants varied greatly with respect to previous laser (SLT/ALT) treatment, phakic status, or previous medication history. Comparison between the two measurements was used to determine change in IOP due to the 4-week washout (Table 2).

### 3.3. Publication Bias

**Figure 4:**
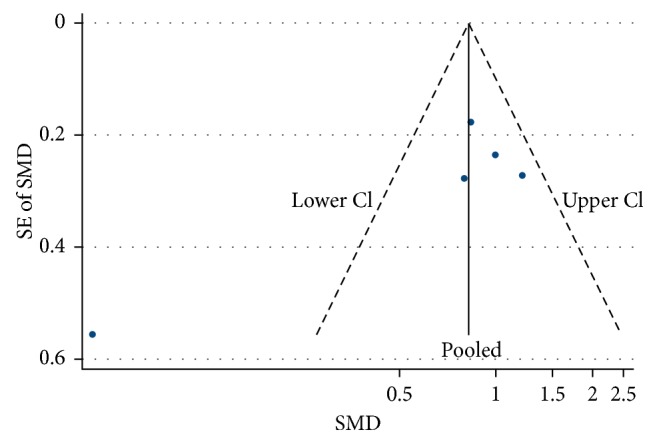
Visual inspection of funnel plot for both pre- and postwashout IOP (Figure 4) did not reveal any asymmetry.

A funnel plot was generated to check publication bias. Visual inspection of the funnel plot for both pre- and postwashout IOP (Figure 4) did not reveal any asymmetry. Additionally, publication bias is only one of the numerous possible explanations for funnel plot asymmetry.

### 3.4. Impact on Primary Outcome

**Figure 5:**
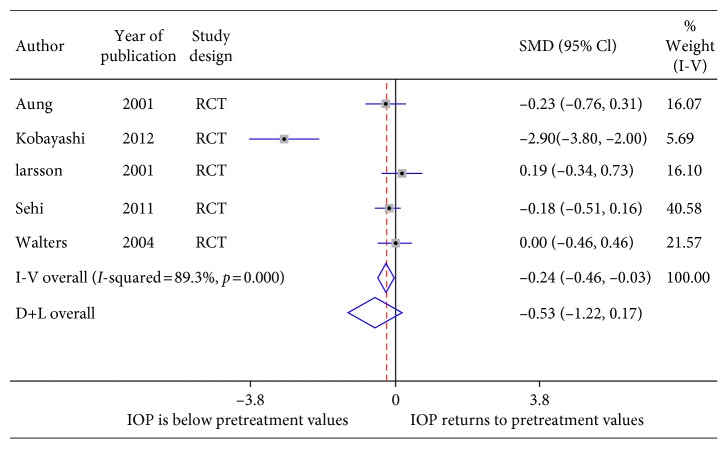
Forest plot of 5 studies of latanoprost washout.

A forest plot was created of five studies which yielded data (Figure 5). Figure 5 summarizes the results for the outcome measure IOP. Five studies (178 subjects) considered the impact on IOP due to discontinuation of topical PGAs in glaucoma or ocular hypertension patients at week four. Two studies showed a sustained IOP-lowering effect relative to pretreatment baseline after four weeks after washout. Heterogeneity between studies that investigated the impact on IOP (*I*^2^ = 89.3%) was significantly (*p*=0.0) high. In studies examining the impact of washout or discontinuation of topical latanoprost (SMD = −0.53, CI = −1.22, 0.17), IOP change was not different as compared to baseline.

### 3.5. Analysis

This study reviewed 548 papers of which 56 met the study criteria. We found that the washout duration between studies varied from 4-5 days to 8 weeks. Seven of the fifty-six articles had a washout duration which was less than 4 weeks, ranging between 4-5 days and 3 weeks. Their year of publication ranged between 1996 and 2015, demonstrating no apparent standard duration of washout of PGA in the setting of clinical trials. The mean washout period in the reviewed articles was 4.56 (±1.25) weeks, with the median and mode both being 4 weeks (Figure 3).

Our analysis of eight articles found that the difference between baseline IOP (pretreatment) and postwashout IOP was statistically significant in only 3 studies. Aung et al. [18] discontinued monotherapy PGA prior to starting a crossover trial of either unoprostone and latanoprost. Sit et al. [23] washed out a heterogenous group of patients (some naïve and others on previous therapy) and then administered only with travoprost for 4 weeks. These participants were washed out for 41–63 hours, after which their IOP was re-measured. Meanwhile, the findings of Kobayashi and colleagues showed that after a 4-week discontinuation of latanoprost, patients who had remained on beta-blockers and brinzolamide did not have an expected increase of 25–33% of pretreatment IOP, but only a 15.4% of baseline IOP increase. As expected, the IOP did not return to baseline as patients remained on at least one other IOP-lowering medication.

The remainder of the analyzed studies did not show a statistically significant effect. Larsson et al. [20] showed that treatment naïve OHT patients who were treated with latanoprost for 4 weeks and then washed out for 4 weeks, returned to their pre-treatment IOPs. Linden et al. [21] found that in patients treated with monotherapy latanoprost for at least 6 months, with most greater than 1 year, experienced a significantly lower IOP of 1.3 mmHg compared to baseline measurements following a 14-day washout period. Sehi et al. [22] analyzed the washout results from a cohort of patients. A four-week washout showed an IOP of 18.0 mmHg which was lower than untreated baseline of 18.8 mmHg. Stewart et al. [22, 23] had an open label assessment of a four-week latanoprost washout in patients who had previously been treated with various classes of IOP-lowering medications. There were inadequate data in the published data to draw specific conclusions regarding washout duration. Finally, Walters et al. looked at 4-week discontinuation of PGA in patients previously treated with IOP-lowering medication and found no statistically significant change after washout. All participants had already discontinued other ocular medications prior to the study.

In a prospective analysis of 603 patients, Jampel and colleagues showed that the IOP changes caused by removing a medication was significantly less than the historically reported maximal IOP changes observed in monotherapy published trials, following discontinuation of a second or third medication class [14]. This finding highlights the complexity of understanding the true IOP effects of single-agent washout in clinical settings where patients are subject to multiple drug classes.

Of the five articles included in the meta-analysis, all except Kobayashi [19] showed a return to baseline IOP following a washout of 3 or 4 weeks. Participants in the study by Kobayashi [19] continued on their other intraocular medications, while the other studies had participants discontinue their previous drops prior to the start of each study [18–22]. Stewart et al. were the first to identify that there may be a variation in the time to return to baseline IOP following washout in individuals being treated with latanoprost. Moreover, they showed that the washout effect is longer than that of brimonidine. They concluded that latanoprost washout periods were often greater than 4 weeks (Stewart et al. 2000).

In an open-label pilot study, Dubiner and colleagues showed that travoprost can have lasting IOP-lowering effects even up to 84 hours after initial dosing. Subsequently, they studied 34 open-angle glaucoma patients and concluded that IOP-lowering effects were identified up to 44 hours after use of travoprost drops [25]. Sit and colleagues also showed that travoprost IOP lowering persisted up to 63 hours after the final dose [23], while Kurtz and Shemesh found that in a certain group of 20 OHT patients, dosing latanoprost once weekly was noninferior to daily use of the medication. Their results were not statistically different up to 3 months of follow-up [26].

## 4. Discussion

Meta-analysis of existing literature demonstrated that after washout, IOP returned to baseline values, with a maximum of 17% IOP reduction (IOPR%) from baseline occurring at 4-weeks after washout. Of the eight articles which met the study criteria, the difference between baseline IOP (pretreatment) and postwashout IOP was statistically significant in only 3 studies [18, 19, 23]. A 4-week discontinuation of latanoprost in patients who had remained on beta-blockers and brinzolamide did not have an expected increase of 25–33% of pretreatment IOP, but only a 15.4% of baseline IOP increase. As expected, the IOP did not return to baseline, as patients remained on at least one other IOP-lowering medication. This suggests that the effectiveness of PGAs is not as high as expected, especially in the setting of using multiple drug classes [14, 19] (Stewart et al. 2000). It is also possible that full responders, partial responders, and nonresponders to the PGA drug class were pooled in these studies, which the mean IOP values would not adequately reflect.

The analysis reveals that there is no standard for PGA washout in reported studies although 4 weeks appears to be the most common period. The analysis also reveals that the various members of the PGA class may differ in the duration of their effectiveness after washout, with some patients having lingering effects to all of the PGA medications. If inappropriate washout is conducted, lingering effects of a PGA drug may influence conclusions regarding subsequent IOP-lowering interventions. As an example, minimally invasive glaucoma surgeries (MIGS) are being suggested as a treatment option to reduce dependency on medical therapy. Lingering IOP effects following an insufficient washout period may prevent the observation of the true IOP-lowering effect of MIGS. This has relevance not only with respect to potential erroneous conclusions from MIGS clinical trials but also can lead to a false security of the effectiveness of MIGS in the clinical setting, the latter resulting in insufficient monitoring after surgery.

The Ocular Hypertension Study found that to achieve target IOP, nearly 40% of patients had to be on at least two IOP-lowering medications [27]. As such, a large portion of glaucoma and OHT patients are on multiple drug classes to control IOP. The findings of Jampel et al. highlight the complexity of the interactions of the IOP-lowering agents when used in combination [14].

The lingering IOP-lowering effects following an insufficient washout may also influence clinical trials designed to evaluate new drugs and/or devices. In clinical trials designed to study the effectiveness of a new IOP-lowering strategy, treatment naive patients would be preferred to avoid the confounding effects of other current or previous interventions. The results of this meta-analysis revealed a paucity of such studies. Only Larsson et al. [20] studied treatment naive participants, with Sit et al. [23] looking at a mix of treatment naive and previously treated patients. Without a thorough understanding of the impact of previous and current topical antiglaucoma medication use, it is not possible to gauge the true efficacy of adjunctive medications, SLT, and/or surgical interventions such as cataract extraction with or without MIGS.

This review was limited by the number of available studies with published data. Only five studies had washout IOP as the main outcome measured [14, 19, 22, 23], with only one study [19] publishing data that could be analyzed for PGAs. A majority of studies were crossover studies which included a discontinuation period.

Another limitation was the considerable amount of heterogeneity among the five studies examining the impact of washout on IOP. This reflected different study populations, demographics, inclusion/exclusion criteria, study location, washout technique, surgeon's experience, available facilities to perform washout, rates of complications, the year washout was performed, and the year the study was conducted. Of note is that there may exists intra-rater and inter rater IOP measurement differences between the various studies. Random-effect computations showed a nonsignificantly controlled and lowered IOP after washout of latanoprost.

In this meta-analysis the Downs and Black checklist [16] was employed to assess the quality of the included studies. This revealed a significant variation in quality scoring with high-, medium-, and poor-quality studies having been reported. Nevertheless, as only five studies were available for analysis, all were included. This is a recognized but necessary limitation due to the few clinical studies currently available. Meta-analysis of RCTs is influenced by inherent biases in the included articles [28]. For example, a multitude of other factors such as level of education, ethnicity, income status, socioeconomic status, previous ocular and nonocular surgeries, family history, other ocular and nonocular diseases, preoperative and postoperative medications, number of medications and comorbidities (e.g., high blood pressure, diabetes, stroke, heart conditions, etc.) could influence the estimates in the original studies.

The results of this quantitative synthesis of the currently available literature suggest that more studies need to be reported to better understand the optimal role of washout in IOP management and topical glaucoma medication management. Washout periods of medications are important to patients, researchers, and physicians. Accurate dosing is key in maintaining IOP to slow or halt progression of glaucomatous changes. Physicians need to know how often to prescribe medications, whether it is safe to discontinue one class and to have guidance when it is appropriate to stop treatment altogether. Researchers, on the other hand, should know the effects of IOP-lowering agents when investigating new therapies or surgical procedures and devices. If the sustained effect of prostaglandin analogues on IOP are not known, baseline IOP measurements cannot be accurately determined in patients previously treated with this drug class.

This meta-analysis demonstrates very little published evidence exists describing the washout effects of prostaglandin drugs on intraocular pressure. Most of the available data are retrospective and confounded by heterogeneity due to exposure to multiple drug classes. Further, confounding factors such as the phakic state and previous laser trabeculoplasty are rarely addressed. Accepting these limitations, four weeks may be a sufficient washout period for many patients who have been subjected to multiple drug therapy. However, the evidence is inconclusive and insufficient to apply to all patients. To address this gap in knowledge, consideration may be given to conduct a prospective, masked study specifically designed to determine the washout period for patients on monotherapy prostaglandin medications. This will be useful to provide guidance to both clinicians and researchers as to when and how to assess the effects of adjunctive therapies in patients previously exposed to first-line prostaglandin medical management.

## Figures and Tables

**Table 1:** Inclusion and exclusion criteria.

Inclusion	Exclusions
Population of study >18 years of age	Non-English papers
Diagnosis of POAG, OHT	Nonhuman studies
Treatment with prostaglandin analogue or prostamide or combination drug which includes prostaglandin analogue or prostamide	Diagnosis of normotensive glaucoma
	Study design: meta-analyses, opinion papers, reviews

**Table 2:** Pre-and postwashout IOP of prostaglandin analogues in research studies 1997–2012, *n* = 8 studies.

References	Study design	Study location	Medication	*N*	Treatment (weeks)	Washout (weeks)	IOP				
							Baseline		Afterwashout		*P* value
							Mean	SD	Mean	SD	
Aung et al. [18]	RCT	Singapore	Latanoprost	27	4	3	22.8	2.08	22.2	3.12	0.4095
			Unoprostone	29	4	3	24.3	3.23	20.9	2.70	<0.001
Kobayashi et al. [19]	RCT	Japan	Latanoprost	20	13.3 ± 5.6	4	23.6	1.6	19.5	1.2	<0.001
Larsson [20]	RCT	Sweden	Latanoprost	27	4	4	23.6	1.04	23.8	1.04	0.4830
Linden et al. [21]	Prospective case series	Sweden	Latanoprost	26	26–52	2	—	—	—	—	—
Sehi et al. [22]	RCT	US	Latanoprost	68	—	4	18.8	4.7	18.0	4.3	0.3023
Sit et al. [23]	Prospective open label	US	Travoprost	20	—	41–63 (hrs)	21.5	2.9	19.6	2.6	0.0354
Stewart et al. [22, 23]	Prospective open label	US	Latanoprost	17	—	4	—	—	—	—	—
Walters et al. [24]	RCT	US	Latanoprost	36	—	4	23.6	2.1	23.6	0.3	1
			Bimatoprost	37	—	4	24.1	2.6	24.1	0.1	1

RCT: randomized control trial; *P* value: Student's *t* test comparing pre- and postwashout IOP.

## References

[1] Camras C. B., Alm A., Watson P., Stjernschantz J. (1996). Latanoprost, a prostaglandin analog, for glaucoma therapy. Efficacy and safety after 1 year of treatment in 198 patients. Latanoprost study groups. *Ophthalmology*.

[2] Higginbotham E. J., Schuman J. S., Goldberg I. (2002). One-year, randomized study comparing bimatoprost and timolol in glaucoma and ocular hypertension. *Archives of Ophthalmology*.

[3] Canadian Opthalmology Association (2009). Canadian ophthalmological society evidence-based clinical practice guidelines for the management of glaucoma in the adult eye. *Canadian Journal of Ophthalmology*.

[4] Alm A. (1991). PhXA34, a new potent ocular hypotensive drug. *Archives of Ophthalmology*.

[5] Ziai N., Dolan J. W., Kacere R. D., Brubaker R. F. (1993). The effects on aqueous dynamics of PhXA41, a new prostaglandin F2*α* analogue, after topical application in normal and ocular hypertensive human eyes. *Archives of Ophthalmology*.

[6] Alm A., Stjemschantz J. (1995). Effects on intraocular pressure and side effects of 0.005% latanoprost applied once daily, evening or morn- ing: a comparison with timolol. *Ophthalmology*.

[7] Camras C. B. (1996). Comparison of latanoprost and timolol in patients with ocular hypertension and glaucoma: a six-month masked, multicenter trial in the United States. The United States latanoprost study group. *Ophthalmology*.

[8] Mishima H. K., Masuda K., Kitazawa Y., Azuma I. A. M. (1996). A comparison of latanoprost and timolol in primary open-angle glaucoma and ocular hypertension A 12-week study. *Archives of Ophthalmology*.

[9] Watson P., Stjernschantz J., Beck L. (1996). A six-month, randomized, double-masked study comparing latanoprost with timolol in open-angle glaucoma and ocular hypertension. *Ophthalmology*.

[10] Gandolfi S., Simmons S. T., Sturm R., Chen K., VanDenburgh A. M. (2001). Three-month comparison of bimatoprost and latanoprost in patients with glaucoma and ocular hypertension. *Advances in Therapy*.

[11] Noecker R. S., Dirks M. S., Choplin N. T., Bernstein P., Batoosingh A. L., Whitcup S. M. (2003). A six-month randomized clinical trial comparing the intraocular pressure-lowering efficacy of bimatoprost and latanoprost in patients with ocular hypertension or glaucoma. *American Journal of Ophthalmology*.

[12] Stewart W. C., Day D. G., Stewart J. A., Schuhr J., Latham K. E. (2001). The efficacy and safety of latanoprost 0.005% once daily versus brimonidine 0.2% twice daily in open-angle glaucoma or ocular hypertension. *American Journal of Ophthalmology*.

[13] Stewart W. C., Holmes K. T., Johnson M. A. (2001). Washout periods for brimonidine 0.2% and latanoprost 0.005%. *American Journal of Ophthalmology*.

[14] Jampel H. D., Chon B. H., Stamper R. (2014). Effectiveness of intraocular pressure-lowering medication determined by washout. *JAMA Ophthalmology*.

[15] Giuffrè G. (1985). The effects of prostaglandin F2a in the human eye. *Graefe’s Archive for Clinical and Experimental Ophthalmology*.

[16] Downs S. H., Black N. (1998). The feasibility of creating a checklist for the assessment of the methodological quality both of randomised and non-randomised studies of health care interventions. *Journal of Epidemiology and Community Health*.

[17] Malvankar-Mehta M. S., Chen Y., Iordanous Y., Wang W., Hutnik C. (2015). iStent as a solo procedure for glaucoma patients: a systematic review and meta-analysis. *PLoS One*.

[18] Aung T., Chew P. T., Yip C. C. (2001). A randomized double-masked crossover study comparing latanoprost 0.005% with unoprostone 0.12% in patients with primary open-angle glaucoma and ocular hypertension. *American Journal of Ophthalmology*.

[19] Kobayashi H. (2012). Efficacy of single glaucoma medication in combined latanoprost and timolol xe therapy in patients with open-angle glaucoma and ocular hypertension: a discontinuation study. *Journal of Ocular Pharmacology and Therapeutics*.

[20] Larsson L. I. (2001). Intraocular pressure over 24 hours after repeated administration of latanoprost 0.005% or timolol gel-forming solution 0.5% in patients with ocular hypertension. *Ophthalmology*.

[21] Linden C., Nuija E., Alm A. (1997). Effects on IOP restoration and blood-aqueous barrier after long-term treatment with latanoprost in open angle glaucoma and ocular hypertension. *British Journal of Ophthalmology*.

[22] Sehi M., Grewal D. S., Feuer W. J., Greenfield D. S. (2011). The impact of intraocular pressure reduction on retinal ganglion cell function measured using pattern electroretinogram in eyes receiving latanoprost 0.005% versus placebo. *Vision Research*.

[23] Sit A. J., Weinreb R. N., Crowston J. G., Kripke D. F., Liu J. H. K. (2006). Sustained effect of travoprost on diurnal and nocturnal intraocular pressure. *American Journal of Ophthalmology*.

[24] Walters T. R., DuBiner H. B., Carpenter S. P., Khan B., VanDenburgh A. M. (2004). 24-hour IOP control with once-daily bimatoprost, timolol gel-forming solution, or latanoprost: A 1-month, randomized, comparative clinical trial. *Survey of Ophthalmology*.

[25] Dubiner H. B., Sircy M. D., Landry T. (2004). comparison of the diurnal ocular hypotensive efficacy of travoprost and latanoprost over a 44-hour period in patients with elevated intraocular pressure. *Clinical Therapeutics*.

[26] Kurtz S., Shemesh G. (2004). The efficacy and safety of once-daily versus once-weekly latanoprost treatment for increased intraocular pressure. *Journal of Ocular Pharmacology and Therapeutics*.

[27] Kass M., Johnson C. A., Keltner J. L., Miller J. P., Ii R. K. P., Wilson M. R. (1994). The ocular hypertension treatment study. *Journal of Glaucoma*.

[28] Egger M., Smith G. D., Schneider M., Minder C. (1997). Bias in meta-analysis detected by a simple, graphical test. *BMJ*.

